# Correction: Evaluation of novel multifunctional polymeric Schiff bases as anticorrosive agents for the medical grade 316L stainless steel

**DOI:** 10.1039/d6ra90052f

**Published:** 2026-07-06

**Authors:** Aisha Hendy, Jehan El-Nady, Asmaa Nour, Safaa M. Ali, T. M. Tamer, Amal S. I. Ahmed, Rabab Mohamed Abou Shahba, Nazly Hassan

**Affiliations:** a Chemistry Department, Faculty of Science (Girls), Al-Azhar University Nasr City Cairo Egypt; b Electronic Materials Department, Advanced Technology and New Materials Research Institute (ATNMRI), City of Scientific Research and Technological Applications (SRTA-City) New Borg El-Arab City, P. O. Box: 21934 Alexandria Egypt; c Composites and Nanostructured Materials Research Department, Advanced Technology and New Materials Research Institute (ATNMRI), City of Scientific Research and Technological Applications (SRTA-City) New Borg El-Arab City, P. O. Box: 21934 Alexandria Egypt na_hassan12@yahoo.com nhassan@srtacity.sci.eg; d Nucleic Acid Research Department, Genetic Engineering and Biotechnology Research Institute (GEBRI), City of Scientific Research and Technological Applications (SRTA-City) P. O. Box: 21934, New Borg El-Arab City Alexandria Egypt; e Polymer Materials Research Department, Advanced Technology and New Materials Research Institute (ATNMRI), City of Scientific Research and Technological Applications (SRTA-City) New Borg El-Arab City, P. O. Box: 21934 Alexandria Egypt

## Abstract

Correction for ‘Evaluation of novel multifunctional polymeric Schiff bases as anticorrosive agents for the medical grade 316L stainless steel’ by Aisha Hendy *et al.*, *RSC Adv.*, 2026, **16**, 6768–6785, https://doi.org/10.1039/D5RA06705G.

The authors regret that an incorrect version of Fig. 1 was included in the original article. The correct version of Fig. 1 is presented below.

**Fig. 1:**
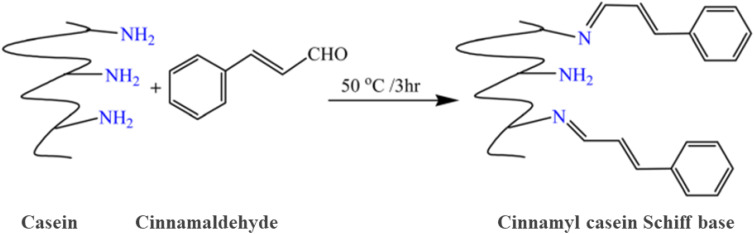
Schematic of the synthesis of casein–cinnamaldehyde Schiff bases.^30^

The Royal Society of Chemistry apologises for these errors and any consequent inconvenience to authors and readers.

